# Markers of erectile dysfunction

**DOI:** 10.4103/0970-1591.42612

**Published:** 2008

**Authors:** Kelvin P. Davies, Arnold Melman

**Affiliations:** Institute of Smooth Muscle Biology and Department of Urology, Albert Einstein College of Medicine, 1300 Morris Park Avenue, Bronx, NY10461, USA

**Keywords:** Biomarker, erectile dysfunction

## Abstract

With the development and marketing of oral pharmacotherapy that is both noninvasive and successful in treating erectile dysfunction (ED), the quest to identify markers of organic ED lost ground. Indeed, the multi-factorial nature of ED may have led many researchers to conclude that searching for a universal marker of ED was futile. However, the realization that ED is strongly correlated with the overall health of men, and may act as a predictor for the development of cardiovascular disease (CVD) and diabetes, has stimulated interest in identifying genes that can distinguish organic ED. In addition, the potential ability to suggest to the patient that ED is reversible (i.e., psychogenic) with a simple test would be of significance to both the physician and patient, as well as for reimbursement issues for therapy by insurance companies. Such a marker may also act as a non-subjective measure of the degree of ED and the efficacy of treatment. This review discusses the importance of identifying such markers and recent work identifying potential markers in human patients.

## INTRODUCTION

A consensus panel of the National Institutes of Health defined erectile dysfunction (ED) as the inability to achieve or maintain an erection sufficient for satisfactory sexual performance. Depending on the cause, ED can be broadly classified as organic, psychogenic, or mixed.[1] The development of ED is generally considered to be multi-factorial and there are several risk factors for ED as outlined in Table 1. However, two of the most common risk factors for organic ED are aging and diabetes.[2] Recent epidemiological studies conducted in the United States found that diabetic men are three times as likely to develop ED as non-diabetic men and men aged 50 to 90 years had a 10-times greater risk for ED than those younger than 50 years of age.[3]

**Table 1:** The major risk factors for the development of erectile dysfunction

Aging	Men who are age 50-90 are 10 times more likely to develop erectile dysfunction than men younger than 50
Comorbidities	Certain medical conditions can increase the risk of erectile dyfunction, including: Diabetes, Cardiovascular disease, Arteriosclerosis (hardening of arteries), Chronic kidney disease, Liver failure, Peyronie's disease (bending of the penis caused by scar tissue), Endocrine disorders, Neurological disorders (such as multiple sclerosis, peripheral neuropathy, stroke), Hypertension (high blood pressure), Psychiatric disorders (such as anxiety, depression, schizophrenia).
Traumatic Conditions	Vascular surgery, Urologic surgery, Pelvic surgeries (particularly for prostate cancer), Spinal cord injury.
Life-style Behavior	Certain behaviors increase the risk of erectile dysfunction, including: Alcohol use, Illegal drug, Anabolic steroid use, Heavy smoking, conflicts with a sexual partner.
Medications	Mecications increasing the risk of erectile dysfunction, include Antihypertensives, Antihypertensives, Antihistamines, Antidepressants, Tranquilizers, Antipsychotics.

A recent study demonstrated that ED not only effects men's sex life, but also significantly effects their overall satisfaction with life in general.[4] Erectile function is increasingly being recognized as an indicator of the overall health of men.[5–7] In particular there is growing recognition that ED is an important marker of vascular disease.[8–11] This realization has led to the recommendation by the American Medical Association that patients with ED should be investigated for cardiovascular disease (CVD).[5,12–16] It is estimated more than 600,000 men aged 40 to 69 years in the United States develop ED and with the availability of effective pharmacotherapy an increasing number of men approach their physicians for treatment. These men who would not otherwise seek medical examination, represent a huge potential for prescreening and prevention of more serious vascular complications.

A recent study by Sun *et al.*,[6] showed that ED was also a significant marker for diabetes, particularly in younger patients. Men 45 years old or younger with ED were more than twice as likely to have diabetes mellitus as men without ED, and men with ED 46 to 65 years old were likely to have diabetes. Thus, markers of ED may represent an early warning for the development of diabetes, which is particularly important considering many diabetic patients remain undiagnosed for several years. As with CVD the pool of patients presenting themselves to physicians for treatment of ED represent a potential for prescreening for diabetes, allowing earlier diagnosis and application of treatments that lower blood glucose thereby reducing the risk of diabetic retinopathy, nephropathy and neuropathy.^17^

In these clinically important scenarios it would be useful to have a biochemical marker that could distinguish between organic and psychogenic ED. It would act as an early warning signal for the development of more serious conditions. Such markers may also act as a non-subjective measure of the degree of ED, and lead to more appropriate dosage treatment regimes. Potentially they could also provide evidence for the efficacy of treatment. This review discusses the ideal characteristics of a marker for ED and recent work identifying potential markers in human patients.

## IDEAL CHARACTERISTICS OF A MARKER FOR ED

There are several characteristics which would make a gene or gene product desirable as a marker for ED. One of these would be that it is changed in all types of etiologies causing ED. This would tend to favor a factor that changes in response to ED, rather than a factor that is involved directly with erectile physiology. Genes that are directly involved in erectile function are more likely to be specific for a particular etiology causing ED.

Ideally, expression of the marker would be proportional to the severity of ED (i.e. moderate, intermediate or severe ED would lead to increasing greater changes in expression of the marker) and respond to the recovery of erectile function following corrective treatments. Potentially this could lead to improved dosage regimens for ED, tailored for individual patients.

The marker would also ideally be present in fluids that could be analyzed by minimally invasive methods, such as blood, saliva or urine. The method of detection should be simple and applicable to a large number of samples. This would ideally be an immunoassay detection system, such as ELISA. Although the expenses for consumable items may be high for the ELISA, this would be compensated by the potential for high sample throughput. Other methods of sample analysis, such as RT-PCR and LC-MS would be limited in the number of samples which could be analyzed. Enzymatic activity could potentially be a cost-effective analytical method allowing high throughput screening. However, relatively few potential markers are enzymes.

These ideal characteristics are shown in Table 2. At present there are no commercially available screens for a marker of ED, despite growing recognition that such a marker would be useful.

**Table 2:** Ideal characteristics of markers for erectile dysfunction

Marker changed in all etiologies resulting in ED
Marker reflects the severity of ED, and efficacy treatments for ED
Marker can be assayed using minimally invasive techniques e.g. blood or saliva
Detectable by immunoassay or other high throughput, cost-effective detection methods

## MICROARRAY STUDIES CAN POTENTIALLY IDENTIFY MARKERS FOR ERECTILE DYSFUNCTION

Several recent studies in rats have used microarray studies to look at global changes in gene expression with onset of ED. Potentially these studies could identify markers of ED, and a comparison of the genes that overlap in these studies would identify genes that are changed in response to several models of ED.

A study by Sullivan *et al.*,[18] looked at changes in gene expression associated with ED after 10 weeks of experimental Type 1 diabetes (streptozotocin-induced diabetic rats compared to littermate controls). Using Affymetrix GeneChip arrays and statistical filtering it was found that 529 transcripts were differentially expressed in the diabetic rat cavernosum compared with control. Gene Ontology (GO) classification indicated that there was a decrease in numerous extracellular matrix genes (e.g., collagen and elastin-related) and an increase in oxidative stress-associated genes in the diabetic rat cavernosum. Some of the differentially expressed genes have a role in vascular dysfunction [e.g., ceruloplasmin (Cp), lipoprotein lipase, and Cd36] or in the modulation of the smooth muscle phenotype (e.g., Kruppel-like factor 5 and chemokine C-X3-C motif ligand 1). These authors concluded that diabetes causes multiple gene changes in the penis resulting in ED. However, another study published by our laboratory using the same STZ-diabetic animal model demonstrated that after one week of diabetes in which there were no significant physiological manifestations of erectile pathology a considerable number of genes (313) were already changed in expression in corporal tissue.[19] Several of the genes identified as changed one week after diabetes, overlapped with the two-month study of Sullivan *et al.* and GO analysis in corpora also showed increases in extracellular matrix genes. Therefore it seems certain that several of the genes changed in expression in erectile tissue in the STZ-diabetic rat model are a response to hyperglycemia. These microarrays also fail to distinguish whether the genes changed in expression have a direct involvement in erectile function, or are indirect and changed in expression as a result of the effect of ED. At present there have been no studies reported for changes in gene expression profiles by microarray in penile tissue in animal models of Type 2 diabetes, despite the far greater prevalence of Type 2 diabetic patients.

Another study using microarray analysis of a different model for ED looked at changes in gene expression associated with hypercholesterolemia.[20] 122 genes were changed in expression in the corpora of hypercholesterolemic rats compared to normal controls. Treatment of ED with a PDE5 inhibitor (udenafil) caused 8 of these genes to return to normal, suggesting that at least 8 of these genes changed as a response to ED, and that the change in expression was reversible once the pathology was treated. This study highlights that only a relatively small number of genes return to their normal expression levels following treatment of ED.

User *et al.*,[21] looked at a rat post-radical prostatectomy model to advance the understanding of neurogenic erectile dysfunction. Five male adult 120-day-old Sprague-Dawley rats underwent bilateral cavernous nerve neurectomy and gene expression was compared to five age-matched controls. One hundred and twenty-six candidate genes were noted to be altered based on the magnitude of expression change using rigorous statistical criteria, including 47 that were down-regulated and 79 that were up-regulated. Although individual genes are not described in this paper, among the many significant changes seen, one dominant class of genes was the submandibular rat genes. The submandibular rat 1 gene (SMR1, now more commonly referred to as Vcsa1) was down-regulated 82.5-fold. Other genes in this family were down-regulated 226 and 90 times. This result was confirmed by reverse transcriptase-polymerase chain reaction and Western blot analyses. The result of this study was supported by work from our group reported recently (and described in detail below) that demonstrated that the same gene was down-regulated in three models of ED.^22^

Earlier studies performed using an array that contained cDNA fragments representing 1176 rat genes looked at gene changes in a traumatic arteriogenic insufficiency rat model.[23] In these studies the pudendal arteries were ligated and microarray analysis was performed at several time points following ligation. The results demonstrated that normal rat corpus cavernosum expressed approximately 200 genes at detectable levels and that ligation produced differential expression of approximately 25 genes, depending on the duration of ligation. The most highly ligation-induced gene was apolipoprotein D (ApoD), with peak expression in the three- and seven-day ligated rats. Three of the insulin-like growth factor binding proteins (IGFBP-1, 3, and 5) were up-regulated in all ligated rats. IGFBP-6, which was one of the most highly expressed genes in the normal corpus cavernosum, was down-regulated in all ligated rats. Cysteine proteases of the cathepsin family were also differentially expressed between control and ligated rats, with cathepsin K being down-regulated most. A few genes were up-regulated only in the six-week ligated rats, including angiotensin-converting enzyme. Finally, VEGF, whose induction has been identified in many other ischemic tissues, was not induced in the corpus cavernous tissue of ligated rats.

These microarray studies illustrate the complexity of gene changes that accompany ED. The studies do not differentiate between genes that have a direct role in erectile function, are an indirect response to ED, or are a response to the condition resulting in ED (such as toxicity associated with STZ, hyperglycemia, trauma, or ischemia). The prohibitive cost of microarray analysis and the necessity of obtaining tissue in order to perform the analysis would prevent the technique being routinely used for patient diagnosis. However, the genes changed in expression in the animal models represent potential markers for patients with ED.

## MARKERS FOR ENDOTHELIAL FUNCTION

**Figure 1:**
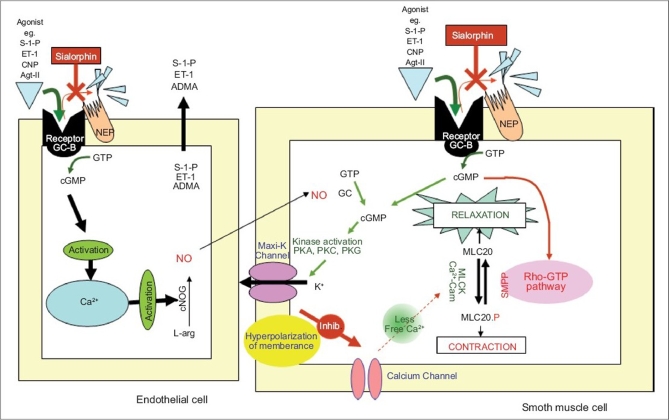
Factors involved in the regulation of corporal smooth muscle tone. Agonists such as Sphingosine-1-phosphate (S-1-P), endothelin-1 (ET-1), C-type natriuretic peptide (CNP) Angiotensin-II (Agt-II) bind to their respective membrane receptor on corporal endothelial and smooth muscle cells and activate downstream signaling pathways most commonly through the mechanism using cyclic guanine monophosphate (cGMP) secondary messenger. Peptide agonists are degraded by neutral endopeptidase (NEP). However, in the presence of sialorphin (or the human homologues which act as NEP inhibitors), these agonists potentially have a prolonged effect, activating downstream mechanisms that result in smooth muscle relaxation. Among these downstream activators is Nitric Oxide (NO) synthesized from L-arginine (L-arg) by nitric oxide synthase (NOS) which is predominantly expressed in the endothelial cells. Nitric oxide diffuses into the corporal smooth muscle cells where it activates guanylate cyclase (GC) contributing to the intracellular pool of cGMP. One of the downstream effects of cGMP is the activation of Maxi-K channels though protein kinases (PK). Efflux of potassium from the cells causes hyperpolarization of the smooth muscle cell membrane, inhibiting influx of Ca^2+^ through calcium channels. Lowered intracellular calcium causes inactivation of myosin light chain kinase (MLCK), thereby promoting smooth muscle relaxation. Activation of the RhoA-GTP pathway inhibits relaxation and results in calcium sensitization. Some markers of endothelial cells, discussed in more detail below, are secreted into the plasma. These include S-1-P, ET-1 and assymetric dimethylarginine (ADMA). GTP = guanosine triphosphate; PKA = protein kinase A; PKC = protein kinase C; PKG = protein kinase G; MLC20 = myosin light chain

The erectile state of the penis depends on the regulating of the tone of smooth muscle (SM) in the walls of the cavernosal arterioles and trabeculae of the cavernosal sinuses. Vasodilation of the blood vessels of the penis resulting in increased blood flow results in an erection. One of the most important mediators of erections is nitric oxide (NO) which is synthesized and released at the endings of the non-adrenergic non-cholinergic (NANC) parasympathetic nerves and by the vascular and sinusoidal endothelial cells. Nitric oxide acts on the SM cells to induce relaxation. Endothelial cells therefore play a critical role in erectile physiology and pathology[24] and has led several groups to investigate biochemical markers of endothelial damage as markers of ED. Indeed the close correlation of ED and endothelial function have led some investigators to coin the phrase “ED2: erectile dysfunction = endothelial dysfunction”.[25] Some of these markers and their relationship with regulating the contractile state of SM cells are shown in Figure 1 and discussed in more detail in the following sections.

### Endothelins

The endothelins (ETs) are a family of 21-amino acid peptides consisting of ET-1, ET-2 and ET-3, each the product of a separate gene and differing from one another by only a few amino acids. They are present in blood, urine, and saliva allowing for noninvasive quantization and can be detected using a commercially available ELISA assay kit.[26] ET-1, the most well-characterized and predominant ET in normal plasma, has been shown to be synthesized by endothelial cells, including corpus cavernosal endothelial cells.[27] The ET-1 peptide is synthesized as a precursor (Big ET-1) and converted to the active peptide by endothelin converting enzyme-1 (ECE-1). ET-1 is a vasoconstrictor and plays an important role as a modulator of erectile function. Endothelin levels in plasma are elevated in the diabetic state in experimental animal models of both Type I and Type II diabetes and the pathways it regulates are also up-regulated.[28] The up-regulation of ET-1 and the genes that it regulates could potentially result in ED because of an increase in cavernossal SM tone.

Studies by Hamed *et al.*,[29] compared plasma levels of ET-1 in patients with ED associated with diabetes (*N* = 12), patients with ED not associated with diabetes (*N* = 12) and patients with psychogenic ED (*N* = 12) to healthy adult men (*N* = 12). Patients with ED showed elevated levels of ET-1 in plasma compared to healthy adults. The elevation of ET-1 was far greater (approximately 10-fold) in patients with organogenic ED disorders compared to the psychogenic disorders. Similar results were obtained by Melegy *et al.*,[30] where they again compared ET-1 levels in patients with organic (both diabetic and non-diabetic patients, N = 16), psychogenic (*N* = 16) and control patients (*N* = 15). In this study the ET-1 was detected at significantly higher levels in the plasma with psychogenic ED compared to control patients. The up-regulation of ET-1 in ED with several etiological causes (including psychogenic causes) suggests that levels are at least partly a response to ED. Evidence for this was recently presented by Aversa *et al.*,[31] where tadafil administration for four weeks to patients with ED (*N* = 20) caused a significant decrease in ET-1 in plasma. Somewhat contradicting the usefulness of ET-1 as a specific marker for ED are studies by Morano *et al.*,[32] described in more detail below) that concluded there were no significant difference in the level of ET-1 between diabetic patients with or without ED, suggesting that ET-1 is a more general marker of diabetes-induced endothelial damage.

### Assymetric dimethylarginine

Another indicator of endothelial cell function is asymmetric dimethylarginine (ADMA), derived from degradation of methylated proteins and found in plasma. Since ADMA levels are about 10-fold higher in endothelial cells compared to other cells, damage to endothelial cells will elevate ADMA serum levels. Several groups have recently attempted to correlate ADMA levels and ED.[33–35] The study by Maas *et al.*,[34] compared three groups of patients 1) men with no ED and no coronary artery disease (CAD) (*N* = 24), 2) men with no ED but with CAD (*N* = 56) and 3) men with ED and CAD. Although there was a trend towards elevated ADMA levels in patients with ED or ED and CAD compared to healthy patients, with this sample size it was not significant. However, in the same paper when men with ED but no CAD (*N* = 76) and men with ED and CAD (*N* = 56) were compared, there was a significant elevation in serum levels of ADMA. Therefore, although ADMA may not be sensitive marker for ED, it may be useful for determining underlying CVD in men with ED. Support for this suggestion can be found in a paper from Wierbicki *et al.*,[33] where 45 men were studied who were suffering from ED. Twenty-four per cent of these men were found to have CAD and treatment was initiated. The remaining patients were treated with sildenafil for 70 days. All patients treated for ED had an improved international index of erectile function (IIEF) score after treatment for 70 days, even though there was no significant change in the ADMA plasma levels. However, patients with CAD had higher levels of ADMA at the beginning of the study, but after 70 days of treatment for CAD these levels were significantly reduced.

There are two methods of analysis of ADMA, immunoassay (ELISA) and direct detection by LC-MS. The ELISA test has been reported as less reliable than LC-MS. Overall, the current evidence suggests that ADMA is useful as a maker for CVD, and may be useful as a secondary screen for CVD in patients presenting with ED. Since ADMA is present in the blood, it can be detected by minimally invasive methods. However, treatment of ED by phosphodiesterase-5 (PDE5) inhibitors does not significantly change the level of ADMA, therefore it would not be useful for monitoring the efficacy of ED treatments.

### Nitric oxide

Nitric oxide is synthesized from L-arginine by a family of nitric oxide sythatases. Although NO is predominantly synthesized in endothelial cells, it plays a well-documented and significant role in vascular SM relaxation by stimulating guanylate cyclase activity. The most commonly prescribed oral erectogenic drugs are phosphodiesterase-5’ inhibitors (PDE5) which act to maintain cGMP levels generated by NO. In studies by El-Latif *et al.*,[36] the plasma levels of NO in 26 diabetic men with ED were compared with 15 impotent men with a psychogenic etiology and 10 control men without ED. There was significantly less NO in the diabetic patients (either on insulin treatment, or not treated) than in the patients with psychogenic ED or control patients. There was a significant correlation between NO and erectile function (determined by pharmaco-penile ultrasonography (PPDU)) in the diabetic group, although the differences in NO levels were not affected by the source of the blood sample (peripheral versus cavernosal) suggesting that the changes are systemic, and not specific to cavernosal tissue.[30] In this study NO levels in diabetic men with ED were compared to diabetic men with erectile function, and therefore it could not be determined if the down-regulation of NO levels may be a general response to diabetes, rather than a response to ED. However, in the study described above by Hamed *et al.*,[29] in patients with mixed etiologic ED disorders NO appeared to be significantly reduced compared to patients without ED.

If studies were performed that confirmed a specific down-regulation of NO levels in response to ED, then several characteristics would favor the use of NO as a marker for ED in a clinical setting. The correlation between NO and erectile function as measured by PPDU suggests levels of NO could determine the severity of ED. The measurement of NO is by a colorometric enzymatic assay by conversion of nitrate to nitrite in the Griess reaction. The reaction can be performed on microtitre plates allowing high-throughput screening of samples.

### MARKERS OF ENDOTHELIAL DAMAGE

Another potential marker for ED is determining the circulating monocyte oxidative activity. The basis of this assay is that diabetes-induced ED is caused by damage to endothelial cells from hyperglycemia and dyslipidemia and similar conditions activate monocytes to produce reactive oxygen species (ROS). Morano *et al.*,[32] compared monocyte oxidative activity by ROS production, and markers of endothelial damage (ET-1, PAI-1 and ICAM-1) in patients with diabetes, with (*N* = 23) or without (*N* = 15) ED. There was no significant difference in the levels of ET-1, PAI-1 between diabetic patients with or without ED, suggesting that these factors are general markers of diabetes-induced endothelial damage. In contrast circulating monocytes from patients with ED showed a significant increase in oxidative activity compared with those without ED. This would suggest that monocyte oxidative activity is a more specific marker of ED than ET-1. However, the difficulty of the assay (isolating monocytes and then using flow cytometry with dihydrorhodamine (DHR) as the oxidative probe) probably will limit the development of using this marker in high-throughput screening.

Another direct marker for endothelial damage is the level of circulating endothelial microparticles (EMP). Elevated numbers of EMP have been found in a number of diseases that have a vascular component. In a recent study by Esposito *et al.*,[37] 30 diabetic patients were compared to 20 age-matched control patients who did not have ED. Diabetic men had a significantly higher number of EMP, and there was a significant correlation between the number of EMP and their IIEF. However, since only diabetic men were used in the test group, alterations in the expression of this marker may be due to diabetes rather than ED. Also, the method of detection which involves preparing platelet-rich plasma, antibody labeling with antigens specific to EMP and the fluorescence-activated cell sorting (FACS) analysis of the particle is probably too cumbersome for routine diagnosis of ED.

### FACTORS INVOLVED IN INFLAMMATION AS MARKERS FOR ERECTILE DYSFUNCTION

The rationale for assaying inflammatory factors for ED is that they have been shown to promote endothelial dysfunction,[38,39] which as described above is an important determinant of erectile function. The connection between inflammation, metabolic syndrome, ED and CAD was recently reviewed by Vlachopoulos *et al.*[40] In a previous study by the same group[41] a total of 141 men were divided into four groups, those with CAD with ED (*N* = 38) and with CAD and without ED (*N* = 25), and those with no CAD and no ED (*N* = 32), or no CAD and ED (*N* =46). They measured a wide range of inflammatory factors including high sensitivity C-reactive protein (hsCRP), interleukin-6 (IL-6), interleukin 1β (IL-1β) and tumor necrosis factor-a (TNF-αas well as several other prothrombotic markers. They found that the majority of these markers were up-regulated in men with ED, irrespective of the etiology resulting in ED. The levels were further elevated in patients with CAD, suggesting the levels corresponded to the degree of vascular disease.

### OTHER FACTORS STUDIED AS MARKERS FOR ERECTILE DYSFUNCTION

### RAGE products

Several papers have highlighted an association between ED and the metabolic syndrome[42–44] and it has been speculated that evaluation of plasma levels of endogenous secretory receptor for advanced glycation end products (esRAGE) may represent a potentially useful laboratory marker for ED.

There are at least three theoretical reasons whereby esRAGE could act as a biomarker for ED. Firstly, there is evidence that levels of esRAGE are strongly and inversely associated with subclinical atherosclerosis, a well-known risk factor for ED. Secondly, levels of esRAGE measured in plasma are inversely related to a number of components of the metabolic syndrome, including body mass index, blood pressure parameters, triglycerides, HbA1c, or insulin resistance index. Of note, esRAGE levels are inversely associated to all these parameters each of which being in turn are related to ED. In addition, AGEs have been shown to be elevated in diabetic human penile tissue, leading to the suggestion that there is a direct role for advanced glycation end products in the pathogenesis of ED.[45] The RAGE products can be detected by commercially available antibodies. Further studies are needed to better establish a relationship between RAGE products and ED.

### Testosterone

There is considerable controversy about the importance of androgens in the initiation and maintenance of erectile function, and this subject has been extensively reviewed (some of the more recent reviews include). [46–48] Recent studies suggest it plays a permissive role in erectile Function. Without adequate androgen levels expression of NOS and PDE-5 genes are altered..[49–51] However, the overall consensus appears to be that testosterone plays more of a role in sexual desire, rather than a direct physiological role in ED.[52] Therefore testosterone levels are more likely to be related to psychogenic rather than organic erectile health.

### Sphingosine-1-phosphate

Sphingosine-1-phosphate (S1P) is a biologically active sphingolipid that is generated upon cell activation from membrane phospholipids as part of the sphingomyelin cycle.[53,54] It is stored in red blood cells and in platelets.[55] S1P acts on five types of G-protein-coupled receptors termed S1P1-S1P5 (originally termed EDG (endothelial differentiation genes)).

A recent publication has reported the existence of S1P1, S1P2 and S1P3 receptors in the human corpus cavernosum.[55] Although S1P has a number of cellular effects, it has been shown to cause an increase in vascular tone mediated through the RhoA/ROK pathway (via the S1P2 and S1P3 receptors) and the phospholipase C beta1 and beta3 pathways (via the S1P1/S1P2 receptors).[56] Because of the potential role of S1P in erectile function, and a report showing that it is elevated in the plasma of patients with at least one of the etiologies associated with ED (the plasma of diabetic patients^57^) S1P is being investigated as a potential marker of ED in our laboratory (Melman and DiSanto, personal communication).

### Vcsa1 family of genes

Recently, our group identified a novel marker of ED in humans, hSMR3A.[58] hSMR3A belongs to a family of genes which includes the human homologue, PROL1 and a rat homologue (SMR1, now called Vcsa1).[58–60] Vcsa1 encodes a precursor protein that gives rise to three peptide products.[61] The mature peptide product of Vcsa1 is a pentapeptide called sialorphin. In vivo sialorphin binds to and inhibits the activity of the membrane-anchored neutral endopeptidase (NEP, EC 3.4.24.11).[62] The NEP plays an important role in nervous and peripheral tissues, as it turns off several peptide-signaling events at the cell surface. It has been demonstrated that sialorphin prevents spinal and renal NEP from breaking down two of its physiologically relevant substrates, substance P and Met-enkephalin *in vitro.* We have recently demonstrated that both gene transfer of Vcsa1 and sialorphin can improve erectile function in the aging rat[22,63] suggesting that they play a direct role in the regulating corporal smooth muscle tone. In organ bath studies we confirmed that sialorphin can increase the rate of C-type natriuretic peptide (CNP) relaxation of contracted corporal smooth muscle tissue. These experiments led to our published hypothesis that sialorphin in corporal smooth muscle tissue can induce relaxation through its action as an NEP inhibitor, preventing the breakdown of CNP bound to receptors.[63]

The identification of Vcsa1 as a potential marker for ED was based on preliminary studies described above in which microarray analysis of gene expression in an animal model for ED resulting from radical prostatectomy suggested that one of the most down-regulated genes in the corpora of these animals was Vcsa1.[21] Our group in preliminary studies using microarray also demonstrated that Vcsa1 was one of the most down-regulated genes in an animal model for diabetes-induced ED. We confirmed this observation using quantitative RT-PCR to measure Vcsa1 expression in non-diabetic and one-week and two-month diabetic rats.[22] In addition to corporal tissue we also looked at Vcsa1 gene expression in the bladder, urethra, and ureter. The C_t_ values of the samples of interest were compared with bladder from control animals (non-diabetic), which we used as the calibrator tissue. Quantitative RT-PCR demonstrated that after one week of diabetes, there was no significant change in the expression of Vcsa1 compared with non-diabetic animals in bladder, urethra, and ureter, and a slight (50% decrease) change in expression of Vcsa1 in the corpora. The one week time point represents a time point where there are already significant changes in gene expression, but no physiologically significant changes in erectile function as measured by the intracorporal pressure/blood pressure, (ICP/BP). However, after two months, there was an even greater decrease in Vcsa1 expression in the corpora, where there was a 10-fold decrease in transcript. Although there is less expression of Vcsa1 in the bladder, RNA extracted from the bladder also demonstrated a significant decrease in Vcsa1 expression associated with diabetes of approximately 5-fold. At the two month time point animals with diabetes had a significant decrease in ICP/BP measurements which would indicate the animals had ED. Similar measurements correlated a decrease in erectile function and Vcsa1expression in an ageing model of ED. Overall our paper demonstrated a correlation between decreased expression of Vcsa1 in the corpora and erectile function in at least three models of ED.[22]

In humans there are at least three homologues to the Vcsa1 gene (We have presented results demonstrating that hSMR3A, hSMR3B and PROL1).[58,59] hSMR3A was down-regulated in human patients with ED. We compared corporal tissue expression in three patients not reporting ED, with diabetic (*N* =5) and non-diabetic patients with ED (*N* = 5). Despite this small sample size, in the group of patients with ED there were significantly lower levels of hSMR3A expression (>10-fold reduction) both in diabetic and non-diabetic patients. This observation suggests that as in the rat model, hSMR3A is a marker for erectile function resulting from different etiologies.

In an aging rat model for ED, Vcsa1 expression is down-regulated.[22] We have recently shown that treating these rats to restore erectile function using gene therapy (hMaxiK) or tadalifil reverses the down-regulation of Vcsa1. Therefore, potentially hSMR3A will be able to monitor the efficacy of ED treatment in human patients.

Overall, the Vcsa1 family of genes have desirable characteristics as markers for ED. They are down-regulated in several etiologies which result in ED and its expression responds to treatment. Potentially, they can be detected by noninvasive methods (for example, immunoassay in saliva). We are presently designing an ELISA test for the protein product of hSMR3A and hSMR3B, which based on the studies of Wisner *et al.*,[59] may be detectable in both serum and saliva samples.

### CONCLUSION

In the past few years the ability of men to achieve an erection has been identified as a barometer of overall health of men. This has led to increased interest in identifying markers that can distinguish between physiological and psychogenic causes of ED. These markers would then be useful in determining the overall “vascular” health of patients, potentially predicting the onset of diabetes and the efficacy of treatment. However, at present most studies have involved small numbers of patients and a definitive identification of a marker awaits larger clinical trials. Since the importance of a marker for ED has now been recognized, it is only a matter of time before such studies are undertaken.
